# General Public’s Information-Seeking Patterns of Topics Related to Obesity: Google Trends Analysis

**DOI:** 10.2196/20923

**Published:** 2020-08-11

**Authors:** Aditya S Pawar, Sajan Nagpal, Neha Pawar, Lilach O Lerman, Alfonso Eirin

**Affiliations:** 1 Division of Nephrology Mayo Clinic Rochester, MN United States; 2 Divison of Gastroenterology University of Chicago Chicago, IL United States; 3 Department of Anesthesiology and Perioperative Medicine University of Rochester Medical Center Rochester, NY United States

**Keywords:** obesity, normalization, public awareness, infodemiology, infoveillance

## Abstract

**Background:**

Obesity is a major public health challenge, and recent literature sheds light on the concept of “normalization” of obesity.

**Objective:**

We aimed to study the worldwide pattern of web-based information seeking by public on obesity and on its related terms and topics using Google Trends.

**Methods:**

We compared the relative frequency of obesity-related search terms and topics between 2004 and 2019 on Google Trends. The mean relative interest scores for these terms over the 4-year quartiles were compared.

**Results:**

The mean relative interest score of the search term “obesity” consistently decreased with time in all four quartiles (2004-2019), whereas the relative interest scores of the search topics “weight loss” and “abdominal obesity” increased. The topic “weight loss” was popular during the month of January, and its median relative interest score for January was higher than that for other months for the entire study period (*P*<.001). The relative interest score for the search term “obese” decreased over time, whereas those scores for the terms “body positivity” and “self-love” increased after 2013.

**Conclusions:**

Despite a worldwide increase in the prevalence of obesity, its popularity as an internet search term diminished over time. The reason for peaks in months should be explored and applied to the awareness campaigns for better effectiveness. These patterns suggest normalization of obesity in society and a rise of public curiosity about image-related obesity rather than its medical implications and harm.

## Introduction

The prevalence of obesity worldwide has consistently increased in the past decades, with a third of the world population now falling under the category of overweight or obese [1,2]. Importantly, obesity increases the risk of several diseases and health conditions, including hypertension, type 2 diabetes, and cardiovascular disease [3]. Worldwide, organizations are using a multifaceted approach to increase population awareness and foster an environment supporting healthy lifestyle by involving stakeholders such as policy makers, community leaders, health care professionals, and school officials.

An important step in addressing the obesity epidemic is acknowledging it as a problem. However, health surveys among the population with obesity and overweight have shown that a significant number of individuals perceived their weight to be “normal” [4]. Underestimation of excessive body weight, or the “normalization” of obesity, is a concern, as it can undermine a serious public health challenge. Public perception of weight and obesity may be influenced by an increase in their prevalence, and therefore, being overweight and obese may become the new normal. Furthermore, individuals with obesity, especially women are affected by its social implications, including discrimination and weight stigma in various walks of life [5-7]*.* This stigma extends to the health care settings and has been observed among physicians, nurses, medical students, and dietitians [6,8]. Social movements such as “body positivity” and “self-love” encourage inclusive and positive conceptualization of body image [7,9] with the principle to foster acceptance and appreciation of all body shapes, sizes, and appearances [10].

Historically, mass media has had an important role in shaping and influencing public health-related beliefs and behaviors [11,12]. The media interest in obesity has been growing, with frequent discussions and articles in the mass media [13,14]. Whether this trend toward increased mass media interest percolates into the public perception of the growing problem of obesity is currently unknown. In the past years, we have seen a change in the media landscape of health information access; it has shifted from television, radio, and print to digital platforms [15].

Health awareness campaigns have shown to increase information-seeking behavior of public pertaining to the agenda of the campaign. The effectiveness of such campaigns can be evaluated using Google Trends, a website by Google that analyzes the popularity of search queries on Google Search across various regions and languages [16]. Public web-based information-seeking trends related to obesity remain unknown. Using Google Trends as a surrogate for public interest, we aimed to study the worldwide patterns of information-seeking by public on obesity and the related terms and topics over the last 16 years.

## Methods

All data used in this paper are publicly available and did not require an institutional review board approval. Google Trends search criteria are reported in Textbox 1.

**Google Trends search criteria.**

- Access date: April 15, 2020.
- Time period of search: January 1, 2004, to December 31, 2019.
- Search syntax: obesity, obese, weight loss, abdominal obesity, body positivity, self-love.
- Geographic region of search: worldwide.
- Query category: global (web search). All available categories on Google Trends were included.
- Quantification of data: monthly and then divided into 4-year quartiles.

We evaluated search activity of the MeSH (Medical Subject Headings) terms related to obesity (obesity, weight loss, obese, and abdominal obesity) and body image (body positivity and self-love) using Google Trends from January 1, 2004, to December 31, 2019, (n=192 months) worldwide using the method recommended by Nuti et al [17]. To assess the impact of terms related to obesity in our multilingual world, we also explored “search topics,” which include Google Trends searches in different languages such as Spanish, Portuguese, Persian, Ukrainian, and Thai [18]. Search terms and topics were chosen from the National Institute of Diabetes and Digestive and Kidney Disease’s glossary of terms related to obesity, as these words are commonly used when people talk or write about obesity [19]. Google Trends is a public web facility of Google Inc, which has been aggregating data on the Google search queries since 2004 [20]. It analyzes web searches to determine their quantity over a period of time and assigns a number between 0 and 100, which reflects the quantity of searches done for a particular term or topic relative to the total number of searches done on Google. This number does not represent an absolute search volume, but rather a normalized value reflected on a scale from 0 to 100, where 100 is the point of maximum popularity among the search terms or topics over a specified time frame. Relative monthly scores for all search terms and topics are expressed as relative interest scores, which are surrogates for the relative popularity of a particular search term and topic over that time frame.

Additionally, Google Trends provides information on the popularity of search terms and topics based on the geographical region, time, and search-related queries. Google trends excludes certain data, such as duplicate searches and search terms and topics with low volume. It filters out queries with special characters such as apostrophes [21]. Similar application of infoveillance in the investigation of health campaign effectiveness has been described previously [16,22]. Mean relative interest scores of search terms and topics were extracted from Google Trends and compared.

The mean relative interest scores were compared across the 4-year quartiles (Quartile 1, 2, 3, and 4) from January 1, 2004, to December 31, 2019. Means were then compared using the Kruskal-Wallis test for the 4 subgroups, as each had 16 observations. A *P* value <.05 was considered significant.

## Results

### Obesity-Related Search Terms and Topics

Table 1 compares the relative interest scores of the obesity-related search terms and topics.

**Figure 1:**
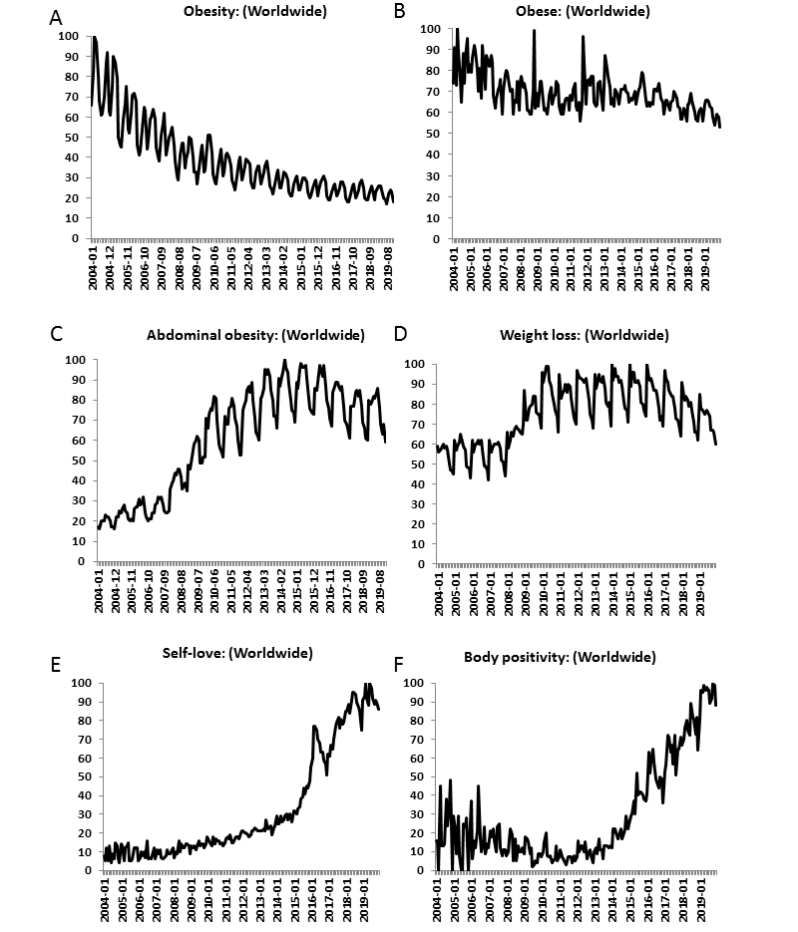
Comparison of the relative interest scores of the obesity-related search terms and topics during the study period.

The mean relative interest score of the search term “obesity” consistently decreased with each quartile (Figure 1A).

**Figure 2:**
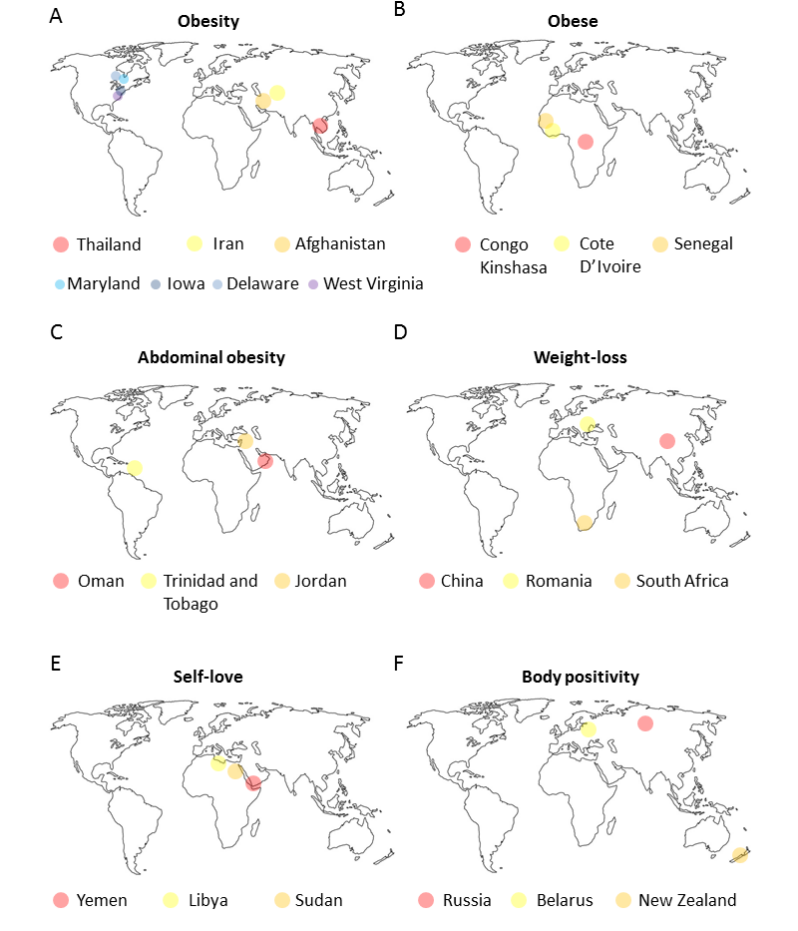
Worldwide distribution of the obesity-related search terms and topics and the top 3 countries with the highest search volume during the study period.

Thailand, Iran, and Afghanistan had the highest search volume during our study period (Figure 2A).

**Table 1:** Relative interest scores of search terms and topics.

Search terms and topics	Quartile 1^a^ Mean (SD)	Quartile 2^b^ Mean (SD)	Quartile 3^c^ Mean (SD)	Quartile 4^d^ Mean (SD)	*P* value
Obesity	75.9 (SD 12.0)	63.7 (SD 6.9)	63.7 (SD 4.7)	61.7 (SD 44.0)	<.001
Weight loss	55.4 (SD 6.2)	78.5 (SD 10.8)	87.4 (SD 8.5)	79.5 (SD 13.1)	<.001
Obese	77.1 (SD 9.9)	67.9 (SD 8.5)	69.8 (SD 5.5)	62.9 (SD 4.9)	<.001
Abdominal obesity	23.7 (SD 4.3)	57.2 (SD 13.9)	83.4 (SD 10.4)	78.6 (SD 10.1)	<.001
Self-love	9.0 (SD 3.3)	14.3 (SD 2.8)	27.1 (SD 7.7)	79.5 (SD 13.1)	<.001
Body-positivity	17.9 (SD 11.8)	9.2 (SD 4.9)	19.7 (SD 11.6)	77.1 (SD 17.6)	<.001

^a^Quartile 1: January 1, 2004, to December 31, 2007.

^b^Quartile 2: January 1, 2008, to December 31, 2011.

^c^Quartile 3: January 1, 2012, to December 31, 2015.

^d^Quartile 4: January 1, 2016, to December 31, 2019.

In the United States (28th country in ranking), highest searches were performed in West Virginia, Maryland, Iowa, and Delaware. The relative interest score for the search term “obese” decreased over time (Figure 1B) with most volume of searches in Congo Kinshasa, Côte D’Ivoire and Senegal (Figure 2B). Contrarily, the relative interest score of “abdominal obesity” increased over time (Figure 1C) with greatest popularity in Oman, Trinidad and Tobago, and Jordan (Figure 2C). The relative interest score of the search topic “weight-loss” consistently increased within the search period with fluctuating popularity. The term appeared to be particularly popular during the month of January. Its median relative interest score for January (n=16) was significantly higher than that for the other months (n=176) during the entire study period (Figure 1D). China, Romania, and South Africa had the most search volumes for the search term “weight loss” (Figure 2D). This study evaluated the queries associated with the obesity-related search topics and terms. Textbox 2 reports the top five queries. There does not seem to be any overlap between the search topics based on the queries, and the reported queries highlight the context behind the search of a particular topic.

**Top 5 queries associated with the obesity-related search topics.**

- Obesity: obesity, 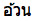 (fat in Thai), obese, obesidad (obesity in Spanish), 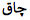 (obese in Persian).
- Obese: obese people, fat, obesity, obese weight, morbidly obese.
- Weight loss: weight loss, lose weight, adelgazar (slim down in Spanish), how to lose weight, emagrecer (lose weight in Portugese).
- Abdominal obesity: fat belly, lose belly fat, how lose belly fat, how to lose belly fat, love handles.
- Self-love: amor (love in Spanish), amor proprio (love self in Spanish, frases amor (phrases love in Spanish), frases (phrases in Spanish), frases amor proprio (phrases self-love in Spanish).
- Body positivity: body, positive body, body positivity, 
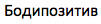
 (body positive in Ukrainian), the body positive.

### Body Positivity–Related Topics

The topics of “Self-love” and “body positivity” have had a consistently increasing interest during the study period (Figure 1E and F). After quartile 3 (2013), there was a steep rise in their relative interest scores. “Body positivity” was most popular in Russia, Belarus, and New Zealand (Figure 2E), whereas “self-love” was most popular in Yemen, Libya, and Sudan (Figure 2F).

## Discussion

As suggested by relative interest scores of the search topics obesity and obese, information-seeking on terms and topics related to obesity on the internet may be declining despite an increase in the prevalence of obesity worldwide. This may indicate rising normalization of obesity in the society. However, this downward trend in searches is limited to obesity as a disease entity, with a contrary increase in search trends and topics related to perception of body image related to obesity, weight loss, and positive acceptance of body image.

Misperception of one’s own weight as normal among the population with overweight and obesity has been described before and termed as normalization of obesity [4]. As one of the major public health challenges worldwide, obesity has significant ramifications on population health with an economic burden on nations, families, and individuals. It poses an additional risk for other diseases and has been well recognized by health professionals as well as public policy makers. Obesity leads to substantial economic impact on medical, productivity, transportation and human capital accumulation cost with reported total annual income loss in excess of US $251 billion in the United States alone [23]. With growing normalization, it is challenging for the public to realize this epidemic and to encourage healthy environment and discussion in addressing obesity. The public needs to be aware of this problem. Medical settings such as health care provider’s office are an ideal place for discussing medical implications of obesity. When done with simple and sensitive language and techniques, these discussions can have positive outcomes and are associated with significant weight loss [24,25]. This approach increases the public curiosity and understanding of medical implications of obesity. The rate of counseling on obesity seems to be declining in the primary care setting [26] especially for patients with obesity and weight-related comorbidities [27]. Primary care providers often have to address several problems within a limited period of time, and weight loss becomes a lower priority [28,29]. Health care professionals are prone to normalizing obesity similar to the general public and may hold a critical negative view of patients with obesity, which leads to fewer interactions with those patients regarding weight management. Further studies are required to explore whether more interactions between medical professionals, patients, and the community about obesity can increase the public interest and internet search activity on obesity, leading to healthy lifestyle and obesity management. Additionally, it is unknown whether normalization of obesity has also affected care providers wherein patients who are overweight and obese might not be counseled because they are considered to be the new normal.

Frequency of internet searches related to weight loss showed an upward trend with peaks in the month of January. This may be due to the end of the holiday season and New Year resolutions. The specific reasons for this increased interest in certain months should be explored and applied to awareness campaigns for better effectiveness. Interestingly, increased peaks in popularity of weight loss also coincide with the search topic of abdominal obesity. It is encouraging that weight loss had the highest volume of search compared to other topics evaluated in this study. A survey of 1000 US adults in November 2017 determined that 45% of Americans share a common New Year’s resolution of weight loss and getting in shape [30]. However, tracking the sustainability of New Year's resolutions over a 2-year period showed that 77% maintained their pledges for 1 week and only 19% for 2 years [31]. Furthermore, public health strategies to tackle weight loss and information sharing on statistics related to obesity seem to correlate with certain peaks in the relative interest scores of weight loss and abdominal obesity [32,33].

Obesity has a well-known psychosocial impact. This impact stems from weight-based discrimination and frequently leads to loss of self-esteem, which may be counterproductive to obesity alleviation. In the 1960s, this led to the body positive movement, with a goal to encourage self-acceptance of one’s own body with an emphasis on self-worth as an individual, rather than on physical appearance [34]. Reassuringly, we found increased search interest in topics involving body positivity and self-love, with a steep rise after year 2013. This may be a result of social media influencers with an emphasis on promotion of body positivity through various outlets [35]. However, social media can be a double-edged sword. It can have a positive impact on body image when consumers imitate role models and accept the help of support groups. However, the same concept can be used for maladaptive body comparison as well [36]. Counseling on obesity and body positivity is a sensitive topic. There is a fine line that differentiates counseling to improve health outcomes in individuals with overweight and obesity from offending them and leading to a worse outcome. Whether intervention from trained weight counselors toward the public would increase public interest in obesity pertaining to medical benefits is currently unknown.

### Limitations

This study is subject to several limitations as with all search trend studies. Currently, Google Trends is the only search engine that offers a data analytics tool, yet Google is the most popular search engine at present. Second, the average user of Google is younger with a higher income and therefore may not be representative of all the population, especially as use of Google requires skills, computer, and internet access. Another limitation of the study is that Google does not provide methodological details of calculating relative search volume. However, we followed methods standardized by Nuti et al [17]. Third, it is difficult to know the intention of an individual searching a particular topic. For example, the topic “fat” could be associated with obesity or as an ingredient in food items. To tackle this problem, we explored related queries to the search topics, which clarify the intentions of the query to some extent; for example, whether they are medical or body image-related. Similarly, this delineates an overlap between the search topics, if any. However, even with the abovementioned limitations, our study has multiple strengths and provides information and generates a hypothesis that the public may be normalizing obesity, and their curiosity towards obesity may be shifting toward the body image more than the negative implications of obesity on health.

### Conclusions

In summary, this study explored the patterns of obesity-related information seeking by general public, suggesting an increase in the normalization of obesity in the society. Furthermore, these data may indicate a shift of search interest from obesity as a medical condition to that as a more body image-related entity. This may have an impact on public health awareness campaigns and may be of interest to policy makers and governments to better address the problem. There is an increased interest in body positivity, which may suggest an increase in positive encouragement in identifying self-worth not based on body weight. It is difficult to delineate whether this stems from increased awareness or negative psychosocial impact leading to curiosity into body positivity. Similarly, it would be helpful for obesity prevention programs to consider these concepts. While more empirical studies are required to characterize these phenomena, the use of Google Trends certainly provides valuable data to assess the public awareness and possibly, health-related campaigns, which are vital to the success of managing obesity at the global level.

## Abbreviations

MeSH: Medical Subject Headings
